# Antecedents and Consequences of Endorsing Prescriptive Views of Active Aging and Altruistic Disengagement

**DOI:** 10.3389/fpsyg.2022.807726

**Published:** 2022-02-01

**Authors:** M. Clara de Paula Couto, Helene H. Fung, Sylvie Graf, Thomas M. Hess, Shyhnan Liou, Jana Nikitin, Klaus Rothermund

**Affiliations:** ^1^Institute of Psychology, Friedrich Schiller University Jena, Jena, Germany; ^2^Department of Psychology, The Chinese University of Hong Kong, Shatin, Hong Kong SAR, China; ^3^Institute of Psychology, Czech Academy of Sciences, Prague, Czechia; ^4^Department of Psychology, North Carolina State University, Raleigh, NC, United States; ^5^Institute of Creative Industries Design, National Cheng Kung University, Tainan, Taiwan; ^6^Department of Developmental and Educational Psychology, University of Vienna, Vienna, Austria

**Keywords:** prescriptive age stereotypes, prescriptive views of aging, expectations about aging, active aging, altruistic disengagement, age differences

## Abstract

In this study, we investigated endorsement of two types of prescriptive views of aging, namely active aging (e.g., prescriptions for older adults to stay fit and healthy and to maintain an active and productive lifestyle) and altruistic disengagement (e.g., prescriptions for older adults to behave altruistically toward the younger generation by granting young people access to positions and resources). The study comprised a large international sample of middle-aged and older adults (*N* = 2,900), covering the age range from 40 to 90 years. Participants rated their personal endorsement of prescriptive views of active aging and altruistic disengagement targeting older adults in general (i.e., “In my personal opinion, older adults should…”). Findings showed that endorsement was higher for prescriptions for active aging than for prescriptions for altruistic disengagement. Age groups in the sample differed regarding their endorsement of both prescriptive views of active aging and altruistic disengagement with older adults showing higher endorsement than middle-aged adults did. Prescriptive views of active aging and altruistic disengagement related positively to each other and to the superordinate social belief that older adults should not become a burden, which attests to their functional similarity. In contrast, prescriptive views of active aging and altruistic disengagement were associated with psychological adjustment in opposite ways, with endorsement of active aging (vs. altruistic disengagement) being related to better (vs. worse) adjustment outcomes such as life satisfaction and subjective health. Our findings highlight the internalization of prescriptive views of aging in older people and their implications for their development and well-being.

## Introduction

Age stereotypes can be defined as beliefs about typical attributes of people of a certain age. For a large part, these beliefs about older adults are dominated by negative attributes (e.g., slow, lonely, helpless, lacking competence; S. Fiske et al., 2002; Kite et al., 2005), but positive perceptions have also been reported (e.g., being experienced or wise; Rothermund et al., 1995). Importantly, age stereotypes have been shown to be incorporated into older adults’ self-concepts (Rothermund and Brandtstädter, 2003; see also Kornadt and Rothermund, 2012; Kornadt et al., 2017), and to influence their development in old age (“stereotype embodiment”; Levy, 2009) by shaping their expectations, motivations, behaviors, and ultimately also their actual experiences and life expectancy (e.g., Levy et al., 2000; Levy, 2009; Voss et al., 2017; Wurm et al., 2017; Kornadt et al., 2019).

Past research has mostly studied attributes typically ascribed to older people, coined descriptive age stereotypes (e.g., Fiske et al., 2002; Rothermund and Brandtstädter, 2003; Kornadt and Rothermund, 2011). More recently, researchers have also investigated expectations people have regarding older people and old age, coined prescriptive age stereotypes (e.g., Martin and North, 2021; but see also Rothermund and Wentura, 2007; North and Fiske, 2013a,b). In contrast to descriptive age stereotypes, which refer to beliefs about how older adults *are*, prescriptive age stereotypes relate to beliefs and expectations about how older adults *should be* and how they *should behave*. One gap in the existing studies on prescriptive age stereotypes is that they have focused on prescriptive beliefs regarding only a specific target characteristic (e.g., that older adults should remain active; Pavlova and Silbereisen, 2012, 2016), without comparing them to prescriptive beliefs relating to other characteristics (e.g., that older adults should be altruistic toward the younger generation, North and Fiske, 2012, 2013a,b; Martin and North, 2021). Such approach has limitations insofar it does not allow for an investigation of whether and how different types of prescriptive beliefs relate to one another.

In the current study, we aim to explore prescriptive stereotypes that target older adults and how age relates to their endorsement. Specifically, we investigate the extent to which people of different ages expect that older adults remain active and engaged (i.e., active aging) and that they pass their access to social resources onto successive generations (i.e., altruistic disengagement). In an age-diverse sample of participants, we seek to explore (a) how different age groups endorse prescriptions for active aging and altruistic disengagement, (b) how these two prescriptive beliefs relate to each other and to a superordinate social belief that older adults should not become a burden to others and to society, and (c) how they relate to relevant outcomes of psychological adjustment. We introduce the term *prescriptive views of aging* to refer to prescriptive beliefs about older adults (i.e., how older adults should be and behave) and distinguish between prescriptive views of active aging and altruistic disengagement.

### Prescriptive Views of Active Aging

Prescriptions for active aging, or activation, refer to the idea that older adults should remain competent and maintain an engaged, active lifestyle. This includes expectations that they continue to be healthy, fit, and mentally sharp, as well as socially and economically engaged (Havighurst, 1961; Rowe and Kahn, 1998; World Health Organization, 2002). This type of prescriptive belief highlights the continuation of societal participation, meaningful activities, and continued fitness and health in old age. Indeed, a qualitative study conducted in Germany showed that the idea that old age was a time to remain active and productive had become more accepted with respect to how age was represented. This idea still coexisted with the more traditional idea that old age is characterized by retirement and withdrawal from activities and social roles, in line with the aforementioned prescriptions for altruistic disengagement (Denninger et al., 2014).

Ekerdt (1986) introduced the concept of “busy ethic” to refer to how retirement age was framed and valued for its active component (i.e., retirement age is socially expected to be an active life period; older adults should remain busy and occupied). Framing old age as a time to remain engaged and active reflects expectations related to activation and fitness, or *prescriptive views of active aging*, with an emphasis on individual responsibility. In terms of their potential social function, we can understand activation as serving the purpose of relieving the social security system in times of demographic aging, which helps maintaining economic sustainability of societies (McNamara et al., 2012; Lessenich, 2015).

Pavlova and Silbereisen (2012, 2016) carried out studies on perceived expectations for active aging and reported that activation demands for older adults had indeed become more prevalent. In addition, perceived activation demands were interpreted as representing a challenge (i.e., potentially entailing the connotation of excitement in pursuing a healthy aging) rather than a threat (i.e., potentially involving stress and anxiety) by most older adults in their sample. Relatedly, perceptions of societal expectations for active aging were shown to be associated with psychologically beneficial outcomes (e.g., positive self-concept, positive affect, and low levels of depression) for at least some older individuals. Accordingly, remaining active can be understood as a way of living a life of continued health, fitness, and participation. Regarding how individuals negotiate the demands of active aging, a longitudinal study carried out in Germany showed that control strategies played a role on how individuals dealt with demands to remain active. Specifically, only when individuals believed they could control active aging demands would they then engage in and put efforts to pursue an active old age (Tomasik and Silbereisen, 2014).

### Prescriptive Views of Altruistic Disengagement

With respect to prescriptions for altruistic disengagement, North and Fiske (2012) first put forth the idea that competition for resources triggers intergenerational tensions because people may perceive that valued resources are unequally distributed and that one group receives a bigger share in comparison to the other. Hereof three prescriptive domains for old age arise: (a) older adults should give up important roles for the younger generation (Succession), (b) older adults should use resources only moderately, especially regarding health care and pensions (Consumption), and (c) older adults should behave in line with their own age instead of trying to act like younger people (Identity). Previous studies showed that younger people endorse prescriptive age stereotypes more strongly than older adults (North and Fiske, 2013a) and resent older adults who violate prescriptions for old age. Accordingly, older adults who do not give away their access to valued resources may be perceived as a burden and become a target of hostile ageism (North and Fiske, 2013b). The proposed prescriptive domains of Succession, Consumption, and Identity (North and Fiske, 2012, 2013a,b) can be understood as components of the overarching social expectation that older adults should cede important roles and grant access to resources to the younger generation, therefore supporting generational solidarity (Ward, 2001). They therefore reflect what we denote in this study as *prescriptive views of altruistic disengagement^1^*.

### Age Differences in Endorsement of Prescriptive Views of Aging

Previous studies have made contrary claims regarding age differences in the endorsement of prescriptive views of aging. Specifically, both increasing and decreasing patterns of identification with and endorsement of prescriptive views of aging can be postulated based on internalization theory (Rothermund and Brandtstädter, 2003) and theories of intergenerational conflict (Ng, 1998; North and Fiske, 2013a,b; see also Löckenhoff et al., 2009), respectively. Correspondingly, two patterns are possible: (1) older people would report higher endorsement of prescriptive views of aging than younger people would, or (2) younger people would report higher endorsement of prescriptive views of aging than older people would.

According to the internalization theory (Rothermund and Brandtstädter, 2003; see also Kornadt and Rothermund, 2012), the processes of internalization and projection should lead to higher endorsement of prescriptive views of aging among older adults. As people get old, they tend to internalize expectations for old age and come to believe that they should behave in accordance with what is socially expected from their age group. At the same time, older adults may project their own aging experiences onto the expectations they have about how others of their own age should behave (Krueger, 2000; Rothermund and Brandtstädter, 2003; Kornadt et al., 2017). Both processes should foster endorsement of prescriptive views of aging in older people. Although previous studies investigating internalization and projection mostly focused on the relation between (descriptive) age stereotypes and personalized views of own aging, the internalization theory would predict similar processes of internalization and projection for the endorsement of prescriptive views of aging (i.e., prescriptions for what older people are expected to do).

From the perspective of an intergenerational conflict account of old age and aging (Ng, 1998; Löckenhoff et al., 2009), however, it seems plausible to assume that younger adults should show higher endorsement of prescriptive views of aging based on their motivation to maintain the status quo (North and Fiske, 2013a,b) or to not be blocked from getting ahead by older people (Martin and North, 2021). Because age groups are interdependent in terms of resources, it is important for younger and middle-aged adults that older people conform to prescriptions that tend to prolong older adults’ subordinate role in society (e.g., older people should be generous and make way for the successive generations, see e.g., North and Fiske, 2013a). Studies conducted in the tradition of the intergenerational conflict perspective have focused on prescriptive views of altruistic disengagement and have found that younger people report higher endorsement of altruistic disengagement, and that they evaluate old people who violate prescriptions for altruistic disengagement more negatively than older people do (North and Fiske, 2013a,b). These studies typically compared samples of young and old participants disregarding middle-aged adults as well as those who are transitioning from middle to old age. It is still an open question whether the same pattern of age differences would be found in samples that cover middle-aged and older adults in different life stages (e.g., middle-aged, young–old, and old–old), and whether similar age differences would also be found for prescriptive views of active aging. Hence, in this study we investigate the endorsement of prescriptive views of aging in a sample that covers the age range of 40–90 years, for both prescriptions for activation and altruistic disengagement.

### In(ter)dependence of Prescriptive Views of Active Aging and Altruistic Disengagement

Although one could think of prescriptive views of activation and altruistic disengagement as representing antagonistic prescriptions for older adults, a closer look at both types of prescriptive views of aging may indicate that they also overlap. Particularly, we hypothesize that prescriptive views of activation and altruistic disengagement may be related in the following ways: First, they may tap into the same overarching social belief that older people should not become a burden to others or to society. The social expectation that older adults should not become a burden and therefore relieve the social system can be achieved by (a) having older adults share their resources with and behave altruistically toward younger people (Martin and North, 2021), but also by (b) having older adults internalize the idea that they should remain competent and fit and therefore holding them responsible for themselves (Denninger et al., 2014; Katz and Calasanti, 2015). In this regard, prescriptive views of activation and altruistic disengagement serve the same social function, setting standards for age-appropriate behavior, both agreeing with the purpose of maintaining social sustainability in the face of a rapidly aging population.

In contrast, prescriptions for active aging and altruistic disengagement may be differently related to important markers of psychological adjustment. In a previous study, Pavlova and Silbereisen (2016) examined the association between societal demands for active aging and psychological adjustment (e.g., well-being, depression) in a sample of older adults. Their findings indicated that perceived activation demands predicted better psychological adjustment only among those older participants who were not involved in formal productive roles (e.g., paid work and volunteering), what the authors referred to as the compensation hypothesis. Based on these findings, we hypothesize that the endorsement of prescriptive views of activation would be positively associated with psychological adjustment in our sample. With respect to endorsement of prescriptive views of altruistic disengagement, no previous study has so far examined how it relates to psychological correlates. We hypothesize, however, that some of the consequences of endorsing altruistic disengagement and behaving in accordance with that prescription could entail feelings of loneliness and functional impairment due to a disuse of social and personal resources (Bortz, 1982).

### The Present Study

The goal of our research is to examine endorsement of two types of prescriptive views of aging, namely activation and altruistic disengagement, in different age groups. Our study addresses two major gaps in the previous literature:

(1) Endorsement of prescriptive views of aging in a sample covering the age range between 40 and 90 years, since past research mostly covered extreme groups of young and old participants (e.g., North and Fiske, 2013a).
(2) Endorsement of prescriptive views of activation and altruistic disengagement simultaneously since previous studies focused on only one type of prescriptions for old age, either those related to the prescriptive views that older adults should altruistically disengage (North and Fiske, 2012, 2013a,b) or those tapping the idea that older adults should remain active (Pavlova and Silbereisen, 2012, 2016).

## Materials and Methods

### Sample

The sample consisted of *N* = 2,900 participants aged 40–90 years who took part in the Aging as Future study (AAF; Lang et al., 2022). The AAF is a longitudinal, international project that spans more than 10 years. It started in 2009 with follow-up waves in 2014 and 2019. In the current study, we used data of the 2019 wave, which covers the age range from 40 to 90 years, representing transition periods from middle age to old age and from old age to very old age. During these transition periods, age stereotypes become relevant to the self. They are hence of special interest to our study. The sample was divided into five age cohorts: 40–49 years old (*n* = 625, 52.8% female, *M*_*age*_ = 43.96, *SD* = 3.14), 50–59 years old (*n* = 625, 53.8% female, *M*_*age*_ = 54.31, *SD* = 2.98), 60–69 years old (*n* = 599, 56.3% female, *M*_*age*_ = 64.49, *SD* = 2.99), 70–79 years old (*n* = 574, 49.8% female, *M*_*age*_ = 74.13, *SD* = 3.04), and 80–90 years old (*n* = 477, 52.4% female, *M*_*age*_ = 83.61, *SD* = 2.88). The samples in each age cohort were balanced with regard to gender, *X*^2^(4) = 5.09, *p* = 0.278. Because of the heterogeneity of variances in education and income, we report results of the Welch test for the ANOVAs. There were differences in marital status, *X*^2^(4) = 85.57, *p* < 0.001, education, *F*(4,1392.93) = 39.56, *p* < 0.001, and income, *F*(4,1388.55) = 75.38, *p* < 0.001. Table 1 provides an overview of the demographic variables in each age cohort.

**TABLE 1:** Sociodemographic information for the full sample.

Variable	40–49	50–59	60–69	70–79	80–89
Gender (% female)	52.8	53.8	56.3	49.8	52.4
Age, *M* (*SD*)	43.96 (3.14)	54.31 (2.98)	64.49 (2.99)	74.13 (3.04)	83.61 (2.88)
Marital status (% married)	67.4	69.6	72.8	65.0	47.8
Education, *M* (*SD*)*^a^*	5.07 (1.81)	4.53 (1.94)	4.26 (2.11)	4.14 (2.26)	3.58 (2.27)
Income (in Euro), *M* (*SD*)*^b^*	5.04 (1.70)	4.93 (1.92)	4.16 (1.97)	3.76 (1.99)	3.51 (1.89)

*^a^Educational level based on the International Standard Classification of Education [ISCED 2011] ranges from 1 (primary education) to 8 (Doctor or equivalent level);*

*^b^monthly household income after taxes – in Euro ranges from 1 (0–500 Euro) to 8 (>10,000 Euro).*

*Post hoc* tests indicated that people in older subsamples had lower education and income as compared to people in the younger subsamples. Specifically, the two older cohorts (70–79 and 80–90) reported the lowest household income and the lowest level of education. In addition to that, education and income showed weak but significant positive associations with activation (*r* = 0.07 and *r* = 0.09, respectively, for education and income) and negative associations with altruistic disengagement prescriptive views of aging (*r* = −0.09 and *r* = −0.13, respectively, for education and income; see Table 2). Since level of education and income differed among age cohorts and showed significant associations with prescriptive views of aging, we decided to add these variables as covariates in our analyses to control for potential confounding effects.

**TABLE 2:** Intercorrelation among all variables.

	Activation	Disengagement	No burden	Life satisfaction	Subjective health	Education level	Income
Activation	1	0.727**	0.636**	0.100**	0.080**	0.073**	0.085**
Disengagement		1	0.663**	0.003	−0.064**	−0.088**	−0.127**
No burden			1	−0.014	−0.024	−0.052**	−0.072**
Life satisfaction				1	0.477**	0.173**	0.219**
Subjective health					1	0.262**	0.292**
Education level						1	0.423**
Income							1

No Burden, expectation that older adults should not become a burden. **p < 0.01.

Participants were from the United States (Wake County, NC, *n* = 414), China (Hong Kong, *n* = 523, and Taiwan, *n* = 615), Germany (Jena and Erlangen, *n* = 790), and the Czech Republic (Plzen and Brno, *n* = 558). The samples in each country were balanced with regard to gender and age, and hence did not differ with regard to these variables. We conducted an additional exploratory analysis that included country as a further factor although this was not the focus of our study (see Appendix).

### Procedures

Participants were recruited via mail or telephone based on large databases that we received from local registry offices or from professional marketing agencies (for details about the recruitment procedure, see the Supplementary Materials).

### Measures

The measures used in the current study were part of a larger questionnaire that was developed in German and then translated into Mandarin, English, and Czech for the respective surveys. The questionnaire included a wide range of variables that assessed different aspects of old age and aging, with prescriptive views of aging being one of them.

#### Prescriptive Views of Aging

We assessed endorsement of prescriptive views of aging using a scale that we developed and tested in this study (see the Supplementary Materials). The developed scale included seven items assessing personal endorsement of two types of prescriptive views of aging: *activation* (four items assessing health, fitness, social engagement, and use of technology, e.g., “In my personal opinion, older adults should stay healthy and fit.”) and *altruistic disengagement* (three items assessing succession, identity, and consumption, e.g., “In my personal opinion, older adults should make way for the younger generation by giving up important roles.”). For each item, participants rated the degree of their agreement on a 5-point Likert scale ranging from 1 (“Do not agree”) to 5 (“Strongly agree”); higher values indicated higher endorsement of the item.

The items were generated based on existing empirical literature on prescriptive beliefs that target older adults. We identified two scales used in studies that assessed prescriptive age stereotypes. One of them was developed by North and Fiske (2013a, SIC Scale) with the aim of measuring the prescriptive domains of Succession, Identity, and Consumption. The other scale focuses on perceived activation demands, like expectations that older adults remain socially active and healthy (Pavlova and Silbereisen, 2012), and how individuals perceive changes in such demands within the last 5 years. We therefore developed the disengagement and activation items based on this literature. Our aim was to develop short scales for each prescriptive domain that at the same time would be general enough to cover the relevant contents for the two dimensions of active aging and disengagement. The reason for that was not to pose a strong burden on participants in an already large survey.

Because reliability based on Cronbach’s alpha assumes equal factor loadings (i.e., essential tau equivalence), we quantified effectiveness and quality of factor score estimates by using the factor determinacy index (FDI) and marginal reliability estimates^2^. FDI of the scores was acceptable in the five age cohorts (FDI_*ACT*_ ≥ 0.89 and FDI_*DIS*_ ≥ 0.78). For the activation factor, marginal reliability was 0.82, 0.84, 0.84, 0.79, and 0.82 in the 40–49, 50–59, 60–69, 70–79, and 80–90 age cohorts, respectively. For the altruistic disengagement factor, marginal reliability was 0.70, 0.87, 0.65, 0.61, and 0.73 in the 40–49, 50–59, 60–69, 70–79, and 80–90 age cohorts, respectively. As can be seen, marginal reliability was lower for the altruistic disengagement than for the activation factor. This might be because the altruistic disengagement factor consists of three different items that we developed to cover the somewhat divergent prescriptive domains of succession, consumption, and identity. The prescriptive domain of identity specially diverges in content from the two other domains of succession and consumption.

One of our hypotheses is that endorsement of activation and altruistic disengagement show functional similarity (i.e., they are both related to the expectation that older adults should not become a burden), while also being content specific for the respective type of prescriptive views of aging. To test the predictive validity of the two scales, we selected the following measures as outcome variables to investigate their associations with endorsement of prescriptive views of activation and altruistic disengagement:

#### Expectation That Older Adults Should Not Become a Burden

Participants rated the degree of their agreement with the item “In my personal opinion older adults should not become a burden to others and society” on a 5-point Likert scale ranging from 1 (“Do not agree”) to 5 (“Strongly agree”); higher values indicated greater agreement with the item. Although it is not possible to compute indicators of reliability for a scale consisting of a single item, the item has high face validity, and it showed substantial correlations with important reference variables: it correlates positively with the altruistic disengagement and activation scales (see the “Results” section), and it goes along with a negative attitude toward State support for older adults, and a reduced perception that older adults are discriminated against (*r* = −0.11, and −0.12, respectively), attesting to its psychometric validity.

#### Life Satisfaction

We assessed domain-specific levels of life satisfaction with a questionnaire developed to assess life satisfaction in different life domains (Kornadt and Rothermund, 2011; Voss et al., 2017). The specific life domains included in the questionnaire were family and one’s committed relationships, friendships and acquaintances, independence and autonomy, leisure activities and commitment, personality and life management, finances and dealing with money, work and professional life, physical fitness, mental fitness, appearance, and health. Participants rated their level of life satisfaction in each domain on a 5-point Likert scale ranging from 1 (“very unsatisfied”) to 5 (“very satisfied”). Ratings were aggregated across domains into a life satisfaction scale. Reliability of the scale was 0.88 in the 40–49 age cohort, 0.91 in the 50–59, 60–69, and 70–79 age cohorts, and 0.92 in the 80–90 age cohort.

#### Subjective Health Status

Participants rated their health status with a single item “How would you describe your current state of health?” with a 5-point scale ranging from 1 (“not good at all”) to 5 (“very good”).

#### Covariates

As covariates, we included those sociodemographic variables that differed between age cohorts and correlated with endorsement of prescriptive views of activation and altruistic disengagement. These were education level and income. Education level was coded in accordance with the guidelines provided in the ISCED 2011 (1 = primary education, 2 = lower secondary education, 3 = upper secondary education, 4 = postsecondary non-tertiary education, 5 = short-cycle tertiary education, 6 = bachelor’s or equivalent level, 7 = master’s or equivalent level, 8 = doctoral or equivalent level; UNESCO Institute for Statistics, 2012). Monthly household income after taxes was assessed on an 8-point scale ranging from 1 (0–500 Euro) to 8 (>10,000 Euro).^3^

### Statistical Analyses

#### Measurement Invariance

The comparison of the two scales of prescriptive views of activation and altruistic disengagement across age cohorts was based on a multiple-group confirmatory factor analysis (CFA). In order for these comparisons to be meaningful, we first had to show that the measure assessing prescriptive views of aging was invariant across the five age cohorts included in the study sample. Due to missing values on items assessing activation and disengagement, 38 participants were not included in the analyses (*N* = 2,862). Measurement invariance was tested in a stepwise manner (configural, metric, and scalar) across individuals aged 40–49, 50–59, 60–69, 70–79, and 80–90 years old. When it was not achieved, a close investigation of the modification indices allowed identification of the most non-invariant parameters in each step, which were then gradually released to assess partial invariance (for detailed information about the measurement invariance analyses, see the Supplementary Materials). The resulting model (partial scalar measurement invariance) with all five age cohorts had a good fit, *X*^2^(96) = 298.883, CFI = 0.959, RMSEA = 0.053, and SRMR = 0.042, changes in CFI, RMSEA, and SRMR were below the cut-off point (Chen, 2007).

We estimated latent means for all age cohorts. The mean values of activation and disengagement were fixed to zero in the reference group (i.e., the 40–49 age cohort). For the other four groups (i.e., 50–59, 60–69, 70–79, and 80–90), the deviation from the intercepts in the reference group was estimated. In order to rescale the latent factor scores to the original scale (1–5), we divided the latent factor scores by the number of items in each factor and added the mean of activation and disengagement of the 40–49 age cohort sample (which was the reference group in the model). All means and standard deviations are presented in Table 3. In order to make the tests of our hypotheses more intuitive, we entered the latent factor means as outcomes or predictors in Regression and ANOVA models. This procedure allows us to make use of the results of measurement modeling, keeping only the common variance for the latent factors, while at the same time using straightforward techniques to estimate age differences and to investigate the relation between prescriptive views of aging and criterion variables.

**TABLE 3:** Activation and disengagement means (*SD* in parenthesis) for age cohorts (*N* = 2862).

Age cohort	Activation	Disengagement
40–49	3.36 (0.23)	2.68 (0.29)
50–59	3.42 (0.24)	2.72 (0.31)
60–69	3.44 (0.23)	2.77 (0.23)
70–79	3.42 (0.21)	2.78 (0.11)
80–90	3.43 (0.22)	2.83 (0.16)
Mean	3.41 (0.23)	2.75 (0.24)

*As none of the interactions between the latent factor means and the covariates were significant, means and standard deviations are reported without controlling for covariates.*

#### Multiple Regression Analyses

To further investigate associations of prescriptive views of activation and altruistic disengagement with criterion variables (expectation that older adults should not be a burden, life satisfaction, and subjective health), we carried out a series of multiple regression analyses with activation and altruistic disengagement as independent variables and each criterion variable as the outcome. Age was included as a control variable in these analyses.

## Results

### Age Differences in Endorsement of Prescriptive Views of Activation and Altruistic Disengagement

Partial scalar measurement invariance among age cohorts was established. Scalar measurement invariance indicates that factor loadings and intercepts can be set equal across groups without decreases in model fit; this is a prerequisite for meaningful mean-level comparisons.

**FIGURE 1:**
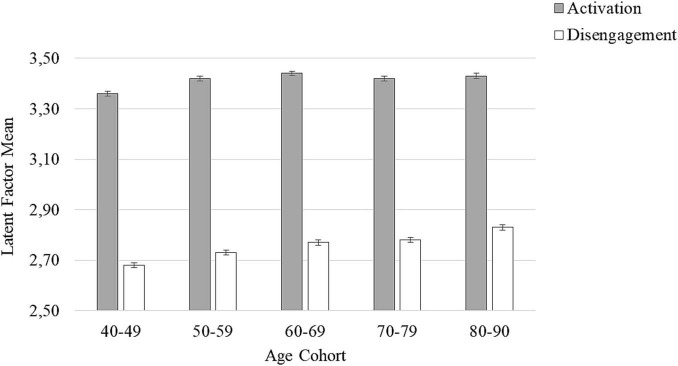
Latent factor means for activation and disengagement by age cohort (40–49, 50–59, 60–69, 70–79, and 80–90 years old), whiskers denote ±1 SE (*N* = 2,862).

In order to compare latent means among age cohorts, a 2 (type of prescription: activation vs. altruistic disengagement) × 5 (age cohort: 40–49 years vs. 50–59 years vs. 60–69 years vs. 70–79 years vs. 80–90 years) factorial ANCOVA with the first factor varying within-participants and the latter varying between-participants was carried out (see Figure 1 for a depiction of average levels of endorsement of the two types of prescriptive views of aging in the different age cohorts). In this analysis, income and education level were included as covariates (see Table 4 for the ANCOVA Summary). This analysis yielded significant main effects for type of prescription, *F*(1,2773) = 42,780.363, *p* < 0.001, $\eta_{\text{p}}^{2}$ = 0.939, and age cohort, *F*(4,2773) = 21.854, *p* < 0.001, $\eta_{\text{p}}^{2}$ = 0.031. These main effects indicated that (a) endorsement of prescriptive views of activation was higher than endorsement of prescriptive views of altruistic disengagement, and (b) endorsement of both types of prescriptive views of aging was higher in the older than younger age groups. Importantly, these main effects were further qualified by a significant two-way interaction of type of prescription with age cohort, *F*(4,2773) = 11.356, *p* < 0.001, $\eta_{\text{p}}^{2}$ = 0.016. This interaction was mainly due to the fact that the endorsement of altruistic disengagement showed a stronger linear increase across age cohorts, *F*(1,2773) = 86.96, *p* < 0.001, $\eta_{\text{p}}^{2}$ = 0.030, compared to the endorsement of activation, *F*(1,2773) = 43.01, *p* < 0.001, $\eta_{\text{p}}^{2}$ = 0.015, thus indicating that the difference in levels of endorsement between activation and altruistic disengagement became smaller with increasing age (see Figure 1). With respect to the covariates, the effects of income and of education were not significant.

**TABLE 4:** GLM ANCOVA summary table for prescription type.

Source	*df*	MS	*F*	*p*	Effect size
Prescription type	1	591.069	42780.363	<0.001	0.939
Prescription type × Education level	1	0.395	28.560	<0.001	0.010
Prescription type × Income	1	1.561	112.972	<0.001	0.039
Prescription type × Age cohort	4	0.157	11.356	<0.001	0.016
Education level	1	0.117	1.251	0.264	0.000
Income	1	0.025	0.266	0.606	0.000
Age cohort	4	2.050	21.854	<0.001	0.031
Within groups	2,773				
Total	2,780				

*MS, mean squares, effect size = $\eta_{\text{p}}^{2}$.*

### Associations of Activation and Altruistic Disengagement With the Expectation That Older Adults Should Not Become a Burden, Life Satisfaction, and Subjective Health

Multiple regression analysis was used to test whether prescriptive views of activation and of altruistic disengagement were significantly associated with participants’ expectation that older adults should not become a burden, their life satisfaction, and their subjective health. In addition to activation and altruistic disengagement, we included age as another independent variable to control for age differences in the criterion variables. The results of the regression analyses are reported in Table 5.

**TABLE 5:** Multiple regression analysis predicting the expectation that older adults should not become a burden, life satisfaction, and subjective health from activation, disengagement, and age (*N* = 2724).

	Older adults should not become a burden				Life satisfaction				Subjective health			
Predictor	*R* ^2^	Beta	*F*	*t*	*R* ^2^	Beta	*F*	*t*	*R* ^2^	Beta	*F*	*t*
	0.498		899.630***		0.021		19.58***		0.100		100.613***	
Constant		−8.41		−34.07***		1.84		10.11***		2.19		8.57***
Activation		0.33		16.47***		0.20		7.41***		0.25		9.34***
Disengagement		0.42		20.88***		−0.14		−5.03***		−0.19		−7.18***
Age		0.06		4.09***		−0.03		−1.42		−0.25		−13.50***

*Standardized beta is reported.*

***p < 0.001.

Endorsement of both prescriptive views of activation and of altruistic disengagement was positively associated with endorsement of the overarching expectation that older adults should not become a burden (activation: β = 0.33, *p* < 0.001, altruistic disengagement: β = 0.42, *p* < 0.001). In line with the finding that activation and altruistic disengagement share a large amount of common variance (see Supplementary Figure 1 and Table 2), this result corroborates our hypothesis that both prescriptive views of activation and of altruistic disengagement are part of the same overarching expectation and possibly fulfill a similar regulatory function that older adults should try to keep their demands on common resources and social welfare systems at a minimum by either behaving altruistically toward the younger generation (e.g., withdrawing from societal positions) or by taking individual responsibility for their lives.

In line with our expectation and replicating the findings by Pavlova and Silbereisen (2016), endorsement of prescriptive views of activation was positively associated with life satisfaction (β = 0.20, *p* < 0.001) and subjective health (β = 0.25, *p* < 0.001). Endorsement of prescriptive views of altruistic disengagement, however, was linked to lower levels of life satisfaction (β = −0.14, *p* < 0.001) and subjective health (β = −0.19, *p* < 0.001).

## Discussion

In this study, we investigated endorsement of prescriptive views of activation and disengagement in a sample of adults covering the age range from 40 to 90 years. For the first time, our findings revealed age differences in endorsement of prescriptions for activation and disengagement in old age. Specifically, the results showed that with increasing age, both prescriptive views of activation and disengagement were more strongly endorsed. Endorsement of activation and disengagement positively correlated with each other and with the overarching social belief that older adults should not become a burden, attesting, as predicted, to the functional similarity of these two prescriptive views of aging. At the same time, endorsement of activation and disengagement were associated with indicators of psychological functioning and adaptation (i.e., life satisfaction and subjective health) in opposite ways, thus making it evident that despite their overlap, these prescriptive views of aging reflect conceptually different facets of prescriptions that target older adults.

### Age Differences in Endorsement of Prescriptive Views of Aging

Our findings show a clear pattern of increasing endorsement of prescriptive views of aging with age, with prescriptions for disengagement being even more strongly endorsed by older adults. This obtained pattern seems to diverge from what has been found in previous studies showing that younger people endorse prescriptive age stereotypes for older adults related to consumption, succession, and identity more strongly than older adults do (North and Fiske, 2013a). There are important methodological aspects that can account for the different findings such as those associated with the measurement scales, like item wording, instructions, format, and how prescriptive views of aging were assessed. Our items on prescriptive views of activation and of altruistic disengagement have a strong emphasis on personal endorsement of prescriptions for older adults in terms of how they should behave. For example, one of our disengagement items reads “In my personal opinion, older adults should use common resources only moderately (e.g., in health care, pensions).” The SIC scale (North and Fiske, 2013a), in contrast, also includes items that describe ageist attitudes toward older adults, purportedly taking the (assumed) perspective of younger people. These items refer to how old people should be treated rather than to how they should behave (e.g., “Doctors spend too much time treating sickly older people.”; “AARP [American Association of Retired Persons] wastes charity money.”; “It is unfair that older people get to vote on issues that will impact younger people much more.”), they highlight intergenerational conflicts explicitly (e.g., “Older people are too big a burden on the healthcare system.”; “Older people are often too much of a burden on families.”), or they express negative descriptive stereotypes about older adults (e.g., “Younger people are usually more productive than older people at their jobs.”). Due to the framing of items in terms of intergenerational conflicts, and by explicitly taking the perspective of people who are not old, the SIC scale may be interpreted differently in terms of their implications and meaning for different age groups and generations, with older adults being prevented from identifying with these statements due to their explicitly ageist, anti-old stance. The framing of our items, in turn, abstained from explicit ageist evaluations and instead highlighted the binding character of prescriptive views of aging, not just toward others (i.e., how others think older people should behave) but also toward oneself (i.e., how one thinks they should behave as an older person). This was done in order to get estimates of the endorsement of these prescriptive views on aging that are unbiased by generational identities and that can be compared across age groups.

Furthermore, the samples employed in the current study and past research were different with respect to age. In our study we investigated an older sample including middle age, old and old–old age (*M*_*age*_ = 62.85, range 40–90 years), while in North and Fiske’s studies, young adults were mainly compared to older adults, leaving out the entire age range of middle adulthood. Thus, in North and Fiske’s (2013b) studies the sampling of extreme age groups allowed for an investigation of endorsement of prescriptive views of aging in the context of intergenerational tensions resulting from competition for scarce resources. We did not include young adults in our sample, and thus we cannot rule out that prescriptive views of disengagement are more pronounced among this age group than among older adults.

Our research yielded evidence for a developmental perspective regarding the endorsement of prescriptive views of aging. Throughout life, everyone must transit from the young to the old group should they live long enough (Rothbaum, 1983; Wentura and Brandtstädter, 2003). How people cope with such a transition over the life course thus becomes a focus of interest. Our findings in a sample that covers the transition period from being a middle-aged adult to being an older person might be particularly sensitive to these changes. With regard to age stereotypes, internalization processes (internalization of age stereotypes, Rothermund and Brandtstädter, 2003; Kornadt and Rothermund, 2012; Kornadt et al., 2017; stereotype embodiment, Levy, 2009) are discussed as resulting from the fact that as one gets old, stereotypes targeting older adults become self-relevant, thus leading to a broad range of consequences (e.g., will to live, Levy et al., 2000; well-being, Rothermund, 2005; life satisfaction, Kornadt and Rothermund, 2011; memory, Levy, 1996). The fact that in our study older adults reported higher endorsement of both activation and disengagement than younger adults suggests that people may internalize these prescriptive views of aging to a greater extent as they transit from adulthood to old age. Therefore, endorsing age-based expectations for old age not only reflects intergenerational tensions as proposed by North and Fiske (2012, 2013a,2013b) but may also reflect internalization of prescriptive views of aging.

Although the overall endorsement of prescriptive views of activation was higher than that of altruistic disengagement, confirming the existence of a focus on the active aging discourse, prescriptive views of disengagement for old age were also endorsed, especially among the older adults. Adults probably expect that older persons withdraw from important roles and positions, thus ceding their resources because this means that younger adults are then the next ones in queue and that they will have their turn in resource access when the time comes (Martin and North, 2021). What may be somewhat surprising is that older persons themselves endorse disengagement prescriptions for old age, even to a larger extent than middle-aged adults do. In addition to an internalization of prescriptive views of aging, projection of one’s own experiences into normative conceptions of old age and aging may also contribute to this finding (cf. Krueger, 2000; Rothermund and Brandtstädter, 2003; Kornadt et al., 2017). Most of the very older adults in our sample will probably have withdrawn from important societal roles (e.g., in the work domain, but also in other domains), and they may tend to interpret their own experience as being normative, further denoting generosity and selflessness, thus justifying their own decisions and experiences.

Moreover, the obtained pattern of increase in endorsement of prescriptive views of altruistic disengagement is in line with findings that showed age differences in altruistic motives. Older adults focus on the greater good and experience self-interest events as less rewarding than prosocial ones (Mayr and Freund, 2020; Isaacowitz et al., 2021; Sparrow et al., 2021). Accordingly, it seems that the shift in older adults’ goals aligns to the social expectations for old age. Whether the shift in older adults’ goals results from an internalization of prescriptive views of aging, or whether the endorsement of these prescriptive views among older adults results from a projection of their changed motivations, or both, remains an open question.

Furthermore, differences between age groups may not just reflect age-related changes in endorsement of prescriptive views of aging but may also originate from younger people actively refraining from endorsing prescriptions for older adults that they might experience as restrictive or prescriptive. Being made aware of the fact that in the future one will be old themselves typically gives rise to more positive attitudes toward aging in young adults (Varkey et al., 2006). In our study this may have prevented especially middle-aged adults from identifying with prescriptions with which they felt uncomfortable because it conflicts with their current situation (e.g., prescriptive views of altruistic disengagement) and would not want to become applied to themselves in the future.

### Functions of Prescriptive Views of Activation and Disengagement

Our findings denote that the prescriptive views of activation and altruistic disengagement coexist and have similar functions in social terms. Activation and altruistic disengagement share a substantial amount of variance, and they both relate positively to the overarching expectation that older adults should not become a burden to others and to society. This highlights that despite being apparently opposite in content, they fulfill a similar overarching motive. On the one hand, older adults shall contribute to overall social welfare by granting access to their roles and resources, that is, by altruistically sharing access to the resources and social roles they have inherited with the younger generation. On the other hand, they also shall contribute to society by alleviating the social welfare system, which can be achieved by taking individual responsibility for remaining active, healthy, and financially independent. One could argue that from the perspective of an older adult, being active may be perceived as something that they are doing for themselves as opposed to subscribing to societal expectations. Having the possibility of extending the duration of adulthood and deferring old age may be an attractive option for many older adults. Our findings, however, showed that both endorsement of prescriptive views of altruistic disengagement and of activation correlated with the expectation that older adults should not become a burden. Hence, even though remaining active may be something older adults strive for and do for themselves, there is an important part of it that is related to fulfilling the societal expectation to not become a burden.

Despite this functional similarity in social terms, activation and altruistic disengagement predicted adaptive outcomes in opposite ways. This indicates that endorsing prescriptive views of activation seems to have positive effects in terms of psychological functioning and adaptation (Menec, 2003; Pavlova and Silbereisen, 2016), while endorsing prescriptive views of altruistic disengagement seems to be detrimental. Accordingly, actual endorsement of altruistic disengagement – and behaving in accordance with this prescriptive view – may affect well-being and health negatively (e.g., focusing only on one’s private life and avoiding social engagement may induce feelings of loneliness, withdrawing from activities and roles may entail functional impairments due to a disuse of capacities; Bortz, 1982). Of course, our cross-sectional data do not allow us to make any causal claims regarding the direction of the association between prescriptive views of aging and outcome variables: Endorsing prescriptive views of aging can be a cause, or an effect of adaptive functioning.

In addition to that, our findings showed that despite the significant associations between prescriptive views of aging and psychological adjustment, these associations were relatively small, and the explained variance was modest. As previous research has found, individuals vary in how they interpret activation demands for old age (Pavlova and Silbereisen, 2012) and in their subjective perceptions of whether they can control such demands (Tomasik and Silbereisen, 2014). Similarly, adhering to or violating prescriptions for altruistic disengagement may have different consequences and implications for psychological adjustment (North and Fiske, 2013b). Hence, it is possible that the association between prescriptive views of aging and psychological adjustment could be stronger when considering moderating variables, such as how individuals interpret prescriptions for old age (e.g., as a threat or as an opportunity) and to what extent they perceive they are able to exert control in order to fulfill such prescriptions.

### Prescriptive Views of Aging: External Demand or Internal Commitment?

With regard to the implications of prescriptive views of activation and altruistic disengagement, it is equally important to discuss the consequences of not conforming to them. For example, North and Fiske’s (2013b) findings about adherence to vs. violation of prescriptions for altruistic disengagement revealed that older adults who violated them were criticized for this type of behavior, hence becoming potential targets of “hostile ageism” instead of the pity-related, paternalistic ageism that often permeates interactions with the older generation (North and Fiske, 2012, 2013b).

When it comes to prescriptive views of activation, Pavlova and Silbereisen (2012) showed that activation demands were associated with appraisals of challenge among highly educated older adults, and with appraisals of threat among disadvantaged ones. Prescriptions for active aging may encourage older adults to remain healthy, socially engaged, and productive, which may be a possibility for some but not for all. It does, however, have a cost, because it puts the focus on the individual and their responsibility to remain competent and active, weakening intergenerational ties or responsibilities, or even leading to intergenerational conflicts when activation prevents altruistic sharing in favor of the younger generation.

Previous research has put a strong emphasis on the demanding character of prescriptive age stereotypes, highlighting the negative implications and sanctions resulting from deviating from what is expected from older adults (see North and Fiske, 2013b, and also Martin et al., 2019). From this perspective, it seems indisputable that prescriptive views of aging lead to social pressure to conform to certain standards and may thus bring about age discrimination and backlash for those older adults who do not adhere to them (because they are not willing to fit the normative standards, or they believe this is not the right way to get old, or they simply cannot achieve the established standards for their age). We do not want to dispute the reality of social prescriptions for old age, non-adherence to which is sanctioned with criticism, exclusion, or other negative consequences.

Our findings, however, mostly tell a somewhat different story, with older adults endorsing prescriptive views of aging, rather than being forced to adhere to prescriptions with which they do not identify. Different motivations can influence old people’s endorsement of prescriptions for old age. Our findings indicate that the desire not to become a burden to others or to society may motivate old people to endorse more specific prescriptive views of aging. We also argue that endorsement of prescriptive views of aging in old people may result from one becoming old themselves and internalizing such prescriptions or from one projecting their own aging experiences onto other old people. Becoming an older adult is a developmental transition that typically involves the adoption of a new personal identity, namely, the identity of an old person. Social identities basically comprise a conception of what it means to be an ideal exemplar of a social group or category (cf. Higgins, 1996; Brandtstädter, 2007). Old age identities mostly reflect idealized images of old persons, depicting them as useful members of society who altruistically pass on their resources, and who actively contribute their share. The negative mirror image of these conceptions comprises images of being or becoming a burden to others or to society. To prevent or counter-act this “feared self” (Markus and Nurius, 1986), old people attempt to reduce their claims on social roles and resources.

There may be, however, other reasons for old people to endorse (or not endorse) age-based prescriptions. For example, old people may internalize prescriptions of active aging so as to reduce their anxiety to become ill, which is driven by the belief that being inactive poses a major threat to the health of older people. Alternatively, they may endorse prescriptive views of aging because of their desire to develop an identity of a “good old person” (i.e., of someone who has aged successfully or as expected) and to avoid being criticized for not acting one’s age. Similarly, a motivation not to become old and to ward off a personal age identification as old might prevent people from endorsing these norms for themselves. Future investigations could examine these and other potential motives that influence old people endorsement (or non-endorsement) of prescriptive views of aging.

In our study, we discuss the social function of prescriptive views of aging and that prescriptions for older adults to stay active and to altruistically disengage both align with the overarching social belief that older people should not become a burden to others or to society. Nonetheless, endorsement of prescriptive views of aging may serve a personal function as well. Prescriptive views of aging reflect knowledge about what life is like when one becomes old, and they convey what it takes to live a good life as an old person. In this respect, prescriptive views of aging specify an identity for older adults. In line with this rationale, endorsement of prescriptive views of active aging and altruistic disengagement could also fulfill a similar personal function. For example, endorsement of prescriptive views of activation and altruistic disengagement could result from older adults’ motivation to set a good example for the next generations by being healthy, independent, and generous, therefore serving as a model of positive aging.

## Limitations

Our study enhances our understanding of prescriptive views of aging, but it is important to emphasize some limitations that should be considered when interpreting our results. First, as already highlighted above, no causal conclusions (e.g., regarding the relation between the endorsement of prescriptive views of aging and criterion variables) are possible as this study relies on cross-sectional data. Second, our cross-sectional design does not allow separating aging effects from cohort effects, which are independent of the process of aging. In this regard, a longitudinal extension of this study would help to better understand the pattern of age differences we have found as well as how internalization and projection processes of prescriptive views of aging unfold across time. Third, we developed the prescriptive views of aging scales of active aging and altruistic disengagement and validated these measures in the same sample that served to test the study hypotheses. However, the sample size of our study is considerably large, implying lower measurement error and more stable factor loadings (Boateng et al., 2018). In spite of that, future studies should further validate the scales in other samples.

Another limitation is that our sample did not include young people but focused on age differences between middle age, old, and old–old age instead. We thus cannot directly compare our results to previous studies that compared young and old samples (North and Fiske, 2013a,b). Including young participants would have allowed us to investigate possible non-linear (e.g., u-shaped) patterns of endorsement of prescriptive views of aging across the life span [with younger people endorsing prescriptive views of aging more strongly than the other age groups as found by North and Fiske (2013a,b)], and to contrast endorsement of prescriptive views of aging with a more developmental account (with internalization and projection shaping increased endorsement with increasing age).

We focused on two types of prescriptive views of aging, altruistic disengagement and activation. Both these prescriptive views relate to the overarching expectation not to become a burden. Although these two prescriptive views of aging cover important prescriptions and expectations for old age, we do not claim that they represent the whole spectrum of expectations and prescriptions for old age. Expectations that older adults should be wise, content, or dignified also target older persons, and future work may examine how endorsement of these prescriptive views of wisdom relates to the other prescriptive views of aging, and whether it predicts positive outcomes in old age. Relatedly, our focus in this study was on the endorsement of general prescriptive views of aging. Assuming that evaluative criteria and behavioral norms differ between life domains (Fiske, 1993), it would be interesting to investigate whether the endorsement of prescriptive views of activation and altruistic disengagement also differs depending on contexts (e.g., activation may dominate for more private domains like leisure, fitness, family, and friends, whereas disengagement might be required in public, or resource-related domains like work, finances, politics, or health care).

In the current study, we assessed endorsement of prescriptive views of aging in a *generalized* way (i.e., “In my personal opinion, older adults should…”). It would be interesting for future studies to also examine *personalized* endorsement of prescriptive views of aging (e.g., “As an older adult, I should…”). Assessing both generalized and personalized endorsement of prescriptive views of aging would allow to investigate internalization processes: Personalized endorsement of prescriptive views of aging may result from endorsing generalized prescriptions for old age, and then internalizing these prescriptions when age identification changes.

As in previous studies (e.g., Pavlova and Silbereisen, 2012; North and Fiske, 2013a), we investigated explicit endorsement of prescriptive views of aging, which was assessed with a self-report measure. Self-report measures constitute a valid methodological approach to the study of prescriptive (and descriptive) beliefs about older people, but they have limitations. They allow for influences of self-presentation and are sensitive to social desirability. In this respect, employing tools that allow assessing endorsement of prescriptive views of aging indirectly is an important step to expand the knowledge about implicit endorsement of this type of age-based prescriptions.

Despite these limitations, our study is the first that systematically tested prescriptive views of activation and altruistic disengagement simultaneously, thus providing further insights on how these two types of prescriptions for old age are related to each other and to different outcome variables. In sum, our findings attest to the existing focus on prescriptive views of activation and how such focus decreases with age as a function of increased endorsement of prescriptive views of altruistic disengagement.

## Conclusion

By focusing on prescriptive views of aging, our study adds a novel element to the large body of research on the important role of views of aging for development (e.g., Levy, 2009; Diehl and Wahl, 2010; Kornadt and Rothermund, 2015). Prescriptive views of aging provide us with a particularly promising perspective on aging and development in old age because prescriptions have a strong social control function and thus can be considered immediate precursors of behavior and preferences for social interaction (Neugarten et al., 1965; North and Fiske, 2013b). Due to their prescriptive character, prescriptive views of aging represent idealized conceptions of living in old age that may be core elements of older adults’ self-concepts and thus can be assumed to be closely related to their thoughts, motivations, intentions, and actions (Ajzen, 1991). Our findings revealed that conceptions of what it means to be a “good old person” (Rothermund, 2019) centered on the general idea of not becoming a burden to others and to society. This very limited conception of the value of the lives of older people is a strong reason to take a critical stance toward both prescriptive views of activation and altruistic disengagement, even though both prescriptions may also capture fundamental truths about living a good life in old age.

## Author’s Note

A preliminary version of this work was presented at the Gerontological Society of America 2019 Annual Scientific Meeting: MCPC and KR (2019). Do not become a burden: Activation and disengagement prescriptive age stereotypes. *Innovation in Aging*, *3* (Suppl. 1), S750. https://doi.org/10.1093/geroni/igz038.2751.

## Data Availability Statement

The raw data supporting the conclusions of this article will be made available by the authors, without undue reservation.

## Ethics Statement

The studies involving human participants were reviewed and approved by Friedrich Schiller University Jena Institutional Review Board (FSV 18/36), Survey and Behavioral Research Ethics Committee, Chinese University of Hong Kong (no protocol #), North Carolina State University Institutional Review Board (project #3425), National Cheng Kung University Governance Framework for Human Research Ethics, and University of Basel, Department of Psychology (019-18-1). The patients/participants provided their written informed consent to participate in this study.

## Author Contributions

MCPC: conceptualization, methodology, data curation and analysis, and writing and editing. HHF, SG, TMH, SL, and JN: methodology, data curation, and editing. KR: conceptualization, methodology, data curation, writing, and editing. All authors contributed to the article and approved the submitted version.

## Conflict of Interest

The authors declare that the research was conducted in the absence of any commercial or financial relationships that could be construed as a potential conflict of interest.

## Publisher’s Note

All claims expressed in this article are solely those of the authors and do not necessarily represent those of their affiliated organizations, or those of the publisher, the editors and the reviewers. Any product that may be evaluated in this article, or claim that may be made by its manufacturer, is not guaranteed or endorsed by the publisher.

## Footnotes

1 We use the term “disengagement” to refer to the expectation that older adults should withdraw from important social roles in order to grant access to resources to the younger generations. We emphasize though that the use of the term disengagement to denote this type of age-related expectation does not imply that we endorse the “disengagement theory” (Cumming and Henry, 1961) and should therefore not be confounded with it.

2 FDI is the correlation between the factor score estimates and the levels on the latent factors they estimate (Beauducel, 2011), and values ≥ 0.80 are considered adequate (Gorsuch, 1983). Marginal reliability is obtained by FDI squared, and it is interpreted as the reliability of the corresponding factor score estimates (Mellenbergh, 1996; Brown and Croudace, 2015).

3 To make the variable comparable across countries, the amount in Euro was then converted in the local currencies according to exchange rates at the time we collected the data (so each category in the scale represents the same absolute value but in different currencies).

## References

[B1] Ajzen I. (1991). The theory of planned behavior. *Organ. Behav. Hum. Decis. Process.* 50 179–211. 10.1016/0749-5978(91)90020-T

[B2] Beauducel A. (2011). Indeterminacy of factor scores in slightly misspecified confirmatory factor models. *J. Mod. Appl. Stat. Methods* 10 583–598. 10.22237/jmasm/1320120900

[B3] Boateng G. O. Neilands T. B. Frongillo E. A. Melgar-Quiñonez H. R. Young S. L. (2018). Best practices for developing and validating scales for health, social, and behavioral research: a primer. *Front. Public Health* 6:149. 10.3389/fpubh.2018.00149 29942800 PMC6004510

[B4] Bortz W. M. (1982). Disuse and aging. *J. Am. Med. Assoc.* 248 1203–1208. 10.1001/jama.1982.03330100041028 7109139

[B5] Brandtstädter J. (2007). “Action perspectives on human development,” in *Theoretical Models of Human Development*, 6 Edn. Vol. 1, ed. Lerner R. M. (New York, NY: Wiley), 516–568.

[B6] Brown A. Croudace T. (2015). “Scoring and estimating score precision using multidimensional IRT,” in *Handbook of Item Response Theory Modeling: Applications to Typical Performance Assessment*, eds Reise S. P. Revicki D. A. (New York, NY: Routledge), 307–333.

[B7] Chen F. F. (2007). Sensitivity of goodness of fit indexes to lack of measurement invariance. *Struct. Equ. Model.* 14 464–504. 10.1080/10705510701301834

[B8] Cumming E. Henry W. (1961). *Growing Old: The Process of Disengagement.* New York, NY: Basic Books.

[B9] Denninger T. van Dyk S. Lessenich S. Richter A. (2014). *Leben im Ruhestand: Zur Neuverhandlung des Alters in der Aktivgesellschaft.* Bielefeld: transcript Verlag.

[B10] Diehl M. Wahl H.-W. (2010). Awareness of age-related change: examination of a (mostly) unexplored concept. *J. Gerontol. Psychol. Sci.* 65 340–350. 10.1093/geronb/gbp110 20008026 PMC2853600

[B11] Ekerdt D. (1986). The busy ethic: moral continuity between work and retirement. *Gerontologist* 26 239–244. 10.1093/geront/26.3.239 3721229

[B12] Fiske A. P. (1993). *Structures of Social Life. The Four Elementary Forms of Human Relations: Communal Sharing, Authority Ranking, Equality Matching, Market Pricing.* New York, NY: Free Press.

[B13] Fiske S. T. Cuddy A. J. C. Glick P. Xu J. (2002). A model of (often mixed) stereotype con- tent: competence and warmth respectively follow from status and competition. *J. Pers. Soc. Psychol.* 82 878–902. 10.1037/0022-3514.82.6.878 12051578

[B14] Gorsuch R. L. (1983). *Factor Analysis.* Hillsdale, NJ: LEA.

[B15] Havighurst R. J. (1961). Successful aging. *Gerontologist* 1 8–13. 10.1093/geront/1.1.8

[B16] Higgins E. T. (1996). “Ideals, oughts, and regulatory focus: affect and motivation from distinct pains and pleasures,” in *The Psychology of Action: Linking Cognition and Motivation to Behavior*, eds Gollwitzer P. M. Bargh J. A. (New York, NY: Guilford Press), 91–114.

[B17] Hu L. Bentler P. M. (1999). Cutoff criteria for fit indexes in covariance structure analysis: conventional criteria versus new alternatives. *Struct. Equ. Model. A Multidiscip. J.* 6 1–55. 10.1080/10705519909540118

[B18] Isaacowitz D. Freund A. Mayr U. Rothermund K. Tobler P. (2021). Age-related changes in the role of social motivation: implications for healthy aging. *J. Gerontol. Ser. B Psychol. Sci. Soc. Sci.* 76 S115–S124. 10.1093/geronb/gbab032 33881524

[B19] Katz S. Calasanti T. (2015). Critical perspectives on successful aging: does it “appeal more than it illuminates”? *Gerontologist* 55 26–33. 10.1093/geront/gnu027 24747713 PMC4986584

[B20] Kite M. E. Stockdale G. D. Whitley B. E. Johnson B. T. (2005). Attitudes toward younger and older adults: an updated meta-analytic review. *J. Soc. Issues* 61 241–266. 10.1111/j.1540-4560.2005.00404.x

[B21] Kornadt A. E. Rothermund K. (2011). Contexts of aging: assessing evaluative age stereotypes in different life domains. *J. Gerontol. Ser. B Psychol. Sci. Soc. Sci.* 66B 547–556. 10.1093/geronb/gbr036 21571702

[B22] Kornadt A. E. Rothermund K. (2012). Internalization of age stereotypes into the self-concept via future self-views: a general model and domain-specific differences. *Psychol. Aging* 27 164–172. 10.1037/a0025110 21875214

[B23] Kornadt A. E. Rothermund K. (2015). “Views on aging: domain-specific approaches and implications for developmental regulation,” in *Annual Review of Gerontology and Geriatrics, 2015: Subjective Aging: New Developments and Future Directions*, Vol. 35, eds Diehl M. Wahl H.-W. (New York, NY: Springer Publishing Co), 121–144.

[B24] Kornadt A. E. Voss P. Rothermund K. (2017). Age stereotypes and self-views revisited: patterns of internalization and projection processes across the life span. *J. Gerontol. Ser. B Psychol. Sci. Soc. Sci.* 72 582–592. 10.1093/geronb/gbv099 26511111

[B25] Kornadt A. E. Voss P. Fung H. Hess T. Rothermund K. (2019). Preparation for old age – the role of cultural context and future perceptions. *J. Gerontol. Psychol. Sci.* 74 609–619. 10.1093/geronb/gby075 29924366

[B26] Krueger J. I. (2000). “The projective perception of the social world: a building block of social comparison processes,” in *Handbook of Social Comparison: Theory and Research*, eds Suls J. Wheeler L. (New York, NY: Plenum Press), 323–351.

[B27] Lang F. R. Lessenich S. Rothermund K. (2022). *Altern als Zukunft – eine Studie der Volkswagenstiftung [Ageing as Future: A Study of the Volkswagen Foundation].* Heidelberg: Springer Spektrum.

[B28] Lessenich S. (2015). “From retirement to active aging: changing images of ‘old age’ in the late twentieth and early twenty-first centuries,” in *Challenges of Aging: Pensions, Retirement and Generational Justice*, ed. Torp C. (Basingstoke: Palgrave Macmillan), 165–177.

[B29] Levy B. (1996). Improving memory in old age through implicit self-stereotyping. *J. Pers. Soc. Psychol.* 71 1092–1107. 10.1037/0022-3514.71.6.1092 8979380

[B30] Levy B. (2009). Stereotype embodiment: a psychosocial approach to aging. *Curr. Dir. Psychol. Sci.* 18 332–336. 10.1111/j.1467-8721.2009.01662.x 20802838 PMC2927354

[B31] Levy B. Ashman O. Dror I. (2000). To be or not to be: the effects of aging stereotypes on the will to live. *Omega* 40 409–420. 10.2190/Y2GE-BVYQ-NF0E-83VR 12557880

[B32] Löckenhoff C. E. De Fruyt F. Terracciano A. McCrae R. R. De Bolle M. Costa P. T. J. (2009). Perceptions of aging across 26 cultures and their culture-level associates. *Psychol. Aging* 24 941–954. 10.1037/a0016901 20025408 PMC2933107

[B33] Markus H. Nurius P. (1986). Possible selves. *Am. Psychol.* 41 954–969. 10.1037/0003-066X.41.9.954

[B34] Martin A. E. North M. S. (2021). Equality for (almost) all: egalitarian advocacy predicts lower endorsement of sexism and racism, but not ageism. *J. Pers. Soc. Psychol.* Advance online publication, 10.1037/pspi0000262 [Epub ahead of print]. 33464112

[B35] Martin A. E. North M. S. Phillips K. W. (2019). Intersectional escape: older women elude agentic prescriptions more than older men. *Pers. Soc. Psychol. Bull.* 45 342–359. 10.1177/0146167218784895 30084290

[B36] Mayr U. Freund A. M. (2020). Do we become more prosocial as we age, and if so, why? *Curr. Dir. Psychol. Sci.* 29 248–254. 10.1177/0963721420910811

[B37] McNamara T. K. Sano J. M. Williamson J. B. (2012). “The pros and cons of pro-work policies and programs for older workers,” in *The Oxford Handbook of Work and Aging*, eds Borman W. C. Hedge J. W. (New York, NY: Oxford University Press), 663–683.

[B38] Mellenbergh G. J. (1996). Measurement precision in test score and item response models. *Psychol. Methods* 1 293–299. 10.1037/1082-989X.9.1.116 15053722

[B39] Menec V. H. (2003). The relation between everyday activities and successful aging: a 6-year longitudinal study. *J. Gerontol. Ser. B* 58 S74–S82. 10.1093/geronb/58.2.S74 12646596

[B40] Neugarten B. L. Moore J. W. Lowe J. C. (1965). Age norms, age constraints, and adult socialization. *Am. J. Sociol.* 70 710–717. 10.1086/223965 14298073

[B41] Ng S. H. (1998). Social psychology in an ageing world: ageism and intergenerational relations. *Asian J. Soc. Psychol.* 1 99–116. 10.1111/1467-839X.00007

[B42] North M. S. Fiske S. T. (2012). An inconvenienced youth? Ageism and its potential intergenerational roots. *Psychol. Bull.* 138 982–997. 10.1037/a0027843 22448913 PMC3838706

[B43] North M. S. Fiske S. T. (2013a). A prescriptive intergenerational-tension ageism scale: succession, identity, and consumption (SIC). *Psychol. Assess.* 25 706–713. 10.1037/a0032367 23544391 PMC3912745

[B44] North M. S. Fiske S. T. (2013b). Act your (old) age: prescriptive, ageist biases over succession, consumption, and identity. *Pers. Soc. Psychol. Bull.* 39 720–734. 10.1177/0146167213480043 23471317 PMC4486053

[B45] Pavlova M. K. Silbereisen R. K. (2012). Perceived level and appraisal of the growing expectations for active ageing among the young-old in Germany. *Res. Aging* 34 80–99. 10.1177/0164027511416371

[B46] Pavlova M. K. Silbereisen R. K. (2016). Perceived expectations for active aging, formal productive roles, and psychological adjustment among the young-old. *Res. Aging* 38 26–50. 10.1177/0164027515573026 25721885

[B47] Rothbaum F. (1983). Aging and age stereotypes. *Soc. Cogn.* 2 171–184. 10.1521/soco.1983.2.2.171

[B48] Rothermund K. (2005). “Effects of age stereotypes on self-views and adaptation,” in *The Adaptive Self. Personal Continuity and Intentional Self-Development*, eds Greve W. Rothermund K. Wentura D. (Göttingen: Hogrefe), 223–242. 10.1037/a0025110

[B49] Rothermund K. (2019). “Was ist ein guter alter Mensch?,” in *Oral Presentation at the 9th IAGG-ER, Gothenburg, Sweden.* Available online at: https://www.youtube.com/watch?v=HHcCZgZMGd0 (accessed October 25).

[B50] Rothermund K. Brandtstädter J. (2003). Age stereotypes and self-views in later life: evaluating rival assumptions. *Int. J. Behav. Dev.* 27 549–554. 10.1080/01650250344000208

[B51] Rothermund K. Wentura D. (2007). “Altersnormen und Altersstereotype [Age norms and age stereotypes],” in *Entwicklung über die Lebensspanne – Ein Lehrbuch [Development over the lifesapn – A textbook]*, eds Brandtstädter J. Lindenberger U. (Stuttgart: Kohlhammer), 540–568. 10.1007/s00391-008-0556-5

[B52] Rothermund K. Wentura D. Brandtstädter J. (1995). Selbstwertschützende Verschiebungen in der Semantik des Begriffs “alt” im höheren Erwachsenenalter [Protecting self-esteem by shifting the semantics of the concept “old” in old age]. *Sprache Kogn.* 14 52–63.

[B53] Rowe J. W. Kahn R. L. (1998). *Successful Aging.* New York, NY: Pantheon/Random House.

[B54] Sparrow E. P. Swirsky L. T. Kudus F. Spaniol J. (2021). Aging and altruism: a meta-analysis. *Psychol. Aging* 36 49–56. 10.1037/pag0000447 33705185

[B55] Tomasik M. J. Silbereisen R. K. (2014). Negotiating the demands of active ageing: longitudinal findings from Germany. *Ageing Soc.* 34 790–819. 10.1017/S0144686X12001304

[B56] UNESCO Institute for Statistics (2012). *International Standard Classification of Education ISCED 2011.* Available online at: http://uis.unesco.org/sites/default/files/documents/international-standard-classification-of-education-isced-2011-en.pdf (accessed October 25).

[B57] Varkey P. Chutka D. S. Lesnick T. G. (2006). The aging game: improving medical students’ attitudes towards caring for the elderly. *J. Am. Med. Dir. Assoc.* 7 224–229. 10.1016/j.jamda.2005.07.009 16698508

[B58] Voss P. Kornadt A. E. Rothermund K. (2017). Getting what you expect? Future self-views predict the valence of life events. *Dev. Psychol.* 53 567–580. 10.1037/dev0000285 28230405

[B59] Ward R. A. (2001). Linkages between family and societal-level intergenerational attitudes. *Res. Aging* 23 179–208. 10.1177/0164027501232003

[B60] Wentura D. Brandtstädter J. (2003). Age stereotypes in younger and older women: analyses of accommodative shifts with a sentence-priming task. *Exp. Psychol.* 50 16–26. 10.1027//1618-3169.50.1.16 12629957

[B61] World Health Organization (2002). *Active Ageing: A Policy Framework.* Geneva: World Health Organization.

[B62] Wurm S. Diehl M. Kornadt A. E. Westerhof G. J. Wahl H.-W. (2017). How do views on aging affect health outcomes in adulthood and late life? Explanations for an established connection. *Dev. Rev.* 46 27–43. 10.1016/j.dr.2017.08.002 33927468 PMC8081396

