# Supplementary material for: Antecedents and Consequences of Endorsing Prescriptive Views of Active Aging and Altruistic Disengagement
Source: Front Psychol. 2022 Feb 1;13:807726. doi: 10.3389/fpsyg.2022.807726 (PMC8844369; doi:10.3389/fpsyg.2022.807726)

**Supplemental Materials**

**Details on Recruitment Procedures**

The recruitment procedure differed slightly depending on the country. Whereas participants in the United States and the Czech Republic were identified using information from marketing firms, contact information was obtained from local registry offices in Germany. In China (Hong Kong and Taiwan), participants were identified either using information from a marketing firm or from the local community. Participants were recruited in several waves in order to obtain samples that were stratified regarding gender and across the targeted age range. Eligible participants were asked for their consent to participate either by telephone or e-mail (China and Czech Republic), by mail (United States, Germany, and Czech Republic), or by personal contact (Czech Republic and Taiwan). Participants answered the questionnaire either alone (United States, China, Germany, and Czech Republic) or with the help of an interviewer due to low literacy or eye problems (Hong Kong, Czech Republic). After completing and returning the questionnaire, participants received approximately a $20 compensation (either as a gift card or in cash). Research procedures were approved by the Institutional Review Boards at Friedrich-Schiller-University Jena, North Carolina State University, Chinese University of Hong Kong, University of Basel, and National Cheng Kung University.

**Prescriptive Views of Aging**

Instructions, items, and response format for the scales assessing endorsement of activation (items 2, 3, 5, 6) and disengagement (items 1, 4, 7) prescriptive views of aging.

Please, indicate **to what degree you believe older adults should correspond to society’s expectations.** Check the box that best describes the degree of agreement with the respective statement.

| **In my personal opinion, older adults should…** | **Do not agree** | **Slightly agree** | **Somewhat agree** | **Mostly agree** | **Strongly agree** |
| --- | --- | --- | --- | --- | --- |
| 1) … make way for the younger generation by giving up important roles (e.g., at work, in politics). | ❑ | ❑ | ❑ | ❑ | ❑ |
| 2) … keep up to date with technology. | ❑ | ❑ | ❑ | ❑ | ❑ |
| 3) … stay healthy and fit (e.g., eat a well-balanced diet, exercise regularly). | ❑ | ❑ | ❑ | ❑ | ❑ |
| 4) … behave like older adults rather than trying to appear young. | ❑ | ❑ | ❑ | ❑ | ❑ |
| 5) … contribute to society as long as possible. | ❑ | ❑ | ❑ | ❑ | ❑ |
| 6) … stay mentally sharp. | ❑ | ❑ | ❑ | ❑ | ❑ |
| 7) … use common resources only moderately (e.g., in health care, pensions). | ❑ | ❑ | ❑ | ❑ | ❑ |

**Measurement invariance analyses across age cohorts**

Measurement invariance was performed as follows, initially, the model fit was assessed cohort-by-cohort. Then, measurement invariance was tested in a stepwise manner (configural, metric, scalar) across the five age cohorts (40-49, 50-59, 60-69-70-79, 80-90). When it was not achieved, a close investigation of the modification indexes allowed identification of the most non-invariant parameters in each step, which were gradually released to assess partial invariance. Measurement invariance was evaluated while considering the recommended cut-off criteria for the change in model fit: ΔCFI ≤ 0.010; ΔRMSEA ≤ 0.015; ΔSRMR ≤ 0.030 (Chen, 2007; Hu & Bentler, 1999). By releasing one factor loading (behavior), partial metric invariance could be considered acceptable [*X*^2^(76) = 267.254, CFI = .964, RMSEA = .056, and SRMR = .040], change in the comparative fit index (CFI), in the root-mean-square error of approximation (RMSEA) and in the standardized root-mean square residual (SRMR) were all within the proposed guidelines. Partial scalar invariance could be considered acceptable as well [*X*^2^(96) = 298.883, CFI = .959, RMSEA = .053, and SRMR = .042] with changes in the CFI, RMSEA and SRMR below the cut-off point (all fit indices are presented in Table S1).

**Cultural Differences: Exploratory Analysis**

Although this was not the focus of our study, we conducted a supplementary exploratory analysis that included culture as an additional factor. This analysis yielded a main effect of culture, *F*(4, 2753) = 97.691, *p* < .001, ηp^2^ = .124, with the highest endorsement of prescriptive views of aging in Taiwan, intermediate endorsement in Hong Kong and in the USA, and the lowest endorsement in the Czech Republic and Germany. The age cohort × culture interaction was also significant, *F*(16, 2753) = 14.827, *p* < .001, ηp^2^ = .079, with endorsement of prescriptive views of aging increasing with age in all cultures except for Taiwan, which showed the reversed pattern, i.e., lower endorsement with age. The two-way interaction type of prescription × culture was significant, *F*(4,2753) = 61.635, *p* < .001, ηp^2^ = .082, with the highest focus on activation in the USA and the lowest in Taiwan. Germany and Hong Kong showed similar levels of focus on activation, which was higher than the one reported in the Czech Republic. The three-way interaction of type of prescription × age cohort × culture was also significant, *F*(16,2753) = 16.901, *p* < .001, ηp^2^ = .089, with the focus on activation following a linear decrease across age cohorts that was the strongest and mostly linear for Germany. Different from the other cultures, in Taiwan, there was a linear increase in the focus on activation with age.

**References**

Chen, F. F. (2007). Sensitivity of goodness of fit indexes to lack of measurement invariance. *Structural Equation Modeling, 14*(3), 464–504. https://doi.org/10.1080/10705510701301834

Hu, L., & Bentler, P. M. (1999). Cutoff criteria for fit indexes in covariance structure analysis: Conventional criteria versus new alternatives. *Structural Equation Modeling: A Multidisciplinary Journal, 6*(1), 1–55. https://doi.org/10.1080/10705519909540118

**Tables**

| Table S1 | | | | |
| --- | --- | --- | --- | --- |
| *Multigroup CFA results. Global fit measures for the exact measurement equivalence of the 7-item model, age cohorts: 40-49, 50-59, 60-69, 70-79, 80-90* | | | | |
|  | Chi2(df) | RMSEA | CFI | SRMR |
| configural | 238.674 (59)*** | 0.059 | 0.968 | 0.033 |
| metric | 376.090 (79)*** | 0.070 | 0.941 | 0.062 |
| partial metric | 267.254 (76)*** | 0.056 | 0.964 | 0.040 |
| scalar | 298.883 (96)*** | 0.053 | 0.959 | 0.042 |
| *Note.* CFA=confirmatory factor analysis; RMSEA= root-mean-square error of approximation; CFI= comparative fit index; *SRMR= standardized root-mean-square residual. *** p <0.001; ** p <0.01; * p < 0.05* | | | | |
|  |  |  |  |  |

**Figures**

**Figure S1.**

Final model structure for age cohort (scalar measurement invariance). The factor loading from dis. to behavior was relaxed. act: activation, dis: disengagement, technol: stay up to date with technology, health: stay healthy and fit, contrib.: contribute to society as long as possible, mental: stay mentally sharp, make place: make way for the younger generation, behavior: behave their own age, resources: use resources moderately. Standardized covariance between independent latent factors: 40-49 = .53, 50-59 = .56; 60-69 = .69; 70-79 =.87, and 80-90 = .89.


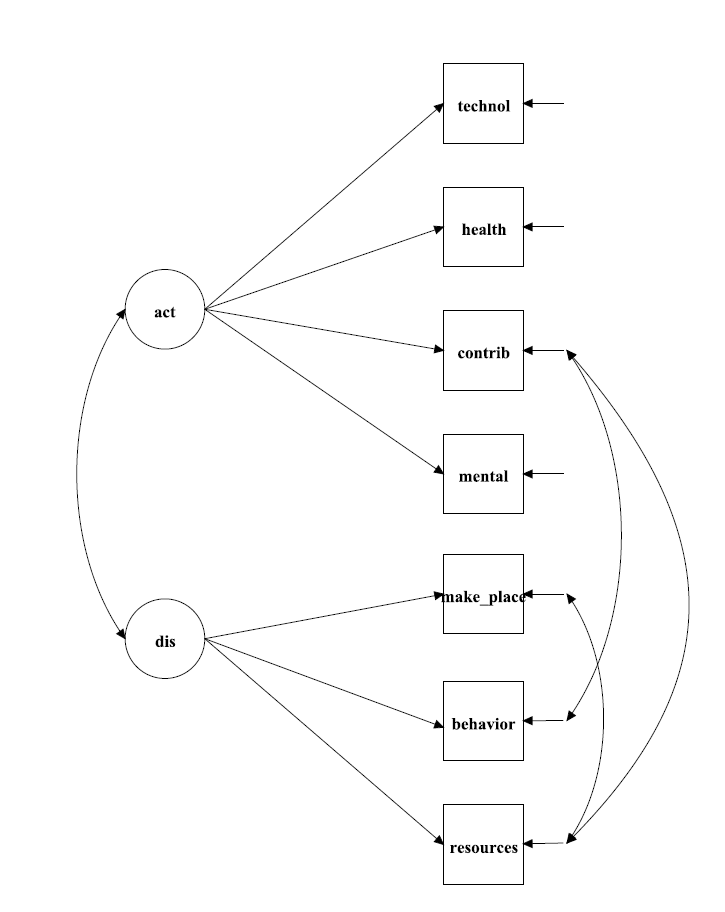

Supplement: Supplementary file 1 [file Data_Sheet_1.docx]
